# Effective structural unit analysis in hexagonal close-packed alloys – reconstruction of parent β microstructures and crystal orientation post-processing analysis

**DOI:** 10.1107/S1600576721011584

**Published:** 2022-02-01

**Authors:** Ruth Birch, Thomas Benjamin Britton

**Affiliations:** a Materials, Imperial College London, London SW7 2AZ, United Kingdom; b Materials Engineering, University of British Columbia, Vancouver, Canada

**Keywords:** electron backscatter diffraction, microstructure, orientation relationships, reconstruction

## Abstract

Materials with an allotropic phase transformation can form microstructures where grains have orientation relationships determined by the transformation history. These microstructures influence the final material properties. In zirconium alloys, there is a solid-state body-centred cubic (b.c.c.) to hexagonal close-packed (h.c.p.) phase transformation, where the crystal orientations of the h.c.p. phase can be related to the parent b.c.c. structure via the Burgers orientation relationship (BOR). In the present work, a reconstruction code, developed for steels and which uses a Markov chain clustering algorithm to analyse electron backscatter diffraction maps, is adapted and applied to the h.c.p./b.c.c. BOR. This algorithm is released as open-source code (via github, as *ParentBOR*). The algorithm enables new post-processing of the original and reconstructed data sets to analyse the variants of the h.c.p. α phase that are present and understand shared crystal planes and shared lattice directions within each parent β grain; it is anticipated that this will assist in understanding the transformation-related deformation properties of the final microstructure. Finally, the *ParentBOR* code is compared with recently released reconstruction codes implemented in *MTEX* to reveal differences and similarities in how the microstructure is described.

## Abstract 2

A method is presented to reconstruct the parent body-centred cubic microstructure from the child hexagonal close-packed microstructure in zirconium alloys. This is used to provide post-processing of the microstructure to understand structural units in the material.

## 1. Introduction

Burgers reported on the body-centred cubic to hexagonal close-packed orientation relationship (OR) in zirconium (Burgers, 1934 ▸) that had been previously reported by Vogel & Tonn (1931 ▸) using X-ray rotation photograph examination (which would now be called a variant of X-ray Laue diffraction) of very large zirconium crystals. In modern materials science, this OR is known as the ‘Burgers orientation relationship’ (BOR). This transformation is important to understand the processing and performance of titanium, zirconium and hafnium alloys, which all have the allotropic phase diagram for high atomic fractions of the base metal in the alloy.

The effect of the presence of related microstructural units on performance is well known and has been reported in the titanium literature, specifically in terms of cold dwell fatigue (Rugg *et al.*, 2007 ▸). Regions of common orientation appear due to the BOR and anisotropy within the strain path which leads to regions of (near) common orientation. Reports of these features vary in the literature, where they are broadly called ‘microtextured regions’ (*e.g.* Cappola *et al.*, 2021 ▸), ‘macrozones’ (*e.g.* Germain *et al.*, 2008 ▸) or the ‘effective structural unit’ (Rugg *et al.*, 2007 ▸). Hafnium and zirconium are less well studied and there are fewer reports in the open literature, but there are limited reasons why similar processing–microstructure–mechanical property linkages will not exist. The only difference is that the loading conditions and safety considerations in their engineering applications will vary.

In practice, the microstructure of the high-temperature phase is often difficult to observe directly. Instead, often the BOR can be observed due to ORs between neighbouring α grains. If the two α grains are from the same parent β grain, then the α–α grain boundary will have a ‘special’ OR. This means that the parent β microstructure can be reconstructed from the α grain structure, provided there is limited subsequent orientation change in the α domain.

To measure the local orientations between grains, electron backscatter diffraction (EBSD) can be used to create 2D (and 3D) microstructure maps where the orientation and phase of each grain are measured at high spatial resolution [for a brief review, see Wilkinson & Britton (2012 ▸)]. These data make it possible to explore the BOR within these engineering alloys.

In a material that has a solid phase transformation, *e.g.* when cooling from a (parent) high-temperature β phase to a (child) lower-temperature α phase, the child phase orientation may be related to the parent grain orientation if an OR is present. For most ORs, the parent phase may have multiple different options for the transformation product orientation, called the variants, due to the symmetry relationships and transformation strain and rotation between the two crystal structures. This is complicated further by the symmetry of the parent crystal and the child crystal which can result in ambiguity in calculating the parent grain orientation from a single child grain. There is a similar challenge in predicting the orientation of the child phase from an initial parent. However, if more than one child orientation is present (*e.g.* as the parent grain contained more than one nucleation site for the phase transformation), then it is possible to reduce the potential candidate parent phases.

The challenge of determining the parent grain orientation using EBSD is well explored in the literature, and many manual (Humbert *et al.*, 1994 ▸, 1995 ▸; Humbert & Gey, 2002 ▸; Tari *et al.*, 2013 ▸), semi-automated (Gey & Humbert, 2003 ▸; Germain *et al.*, 2012 ▸) and fully automated (Cayron, 2007 ▸; Germain *et al.*, 2012 ▸, 2019 ▸; Glavicic *et al.*, 2003*b* ▸; Hadke *et al.*, 2016 ▸; Davies, 2009 ▸; Nyyssönen *et al.*, 2018 ▸) grain reconstruction codes have been developed. Groups of similar grains can be identified using the grain boundary misorientation, the mean grain orientations or the pixel orientations in EBSD data. Refinements to this process include iterating the OR to take into account the experimental results and using more complex grain grouping methods, for example, using the Markov clustering algorithm (van Dongen, 2000 ▸).

Many reconstruction methods derive from ideas shared by Humbert and co-workers (Humbert *et al.*, 1994 ▸, 1995 ▸; Humbert & Gey, 2002 ▸). These methods start by comparing the experimental grain boundary misorientations with the theoretical grain boundary misorientations (between potential α variants) from the OR (Humbert *et al.*, 2015 ▸; Germain *et al.*, 2007 ▸). The aim is to identify the parent grain boundaries (*i.e.* those that do not match the theoretical misorientations between variants), usually as part of a grain grouping step.

A limitation of these methods is often that they do not deal well with experimental deviation from the OR, for example deviations caused by internal stresses from thermal or mechanical processing. Cayron *et al.* (2006 ▸) compared the tolerance for the misorientation of the room-temperature phase versus the theoretical misorientations for the OR in titanium/zirconium alloys and steels, and found these to be ∼5 and ∼2–4°, respectively. To overcome this issue, an iterative step for the OR has been introduced in some cases, such as in work by Nyyssönen *et al.* (2016 ▸, 2018 ▸) and Nyyssönen (2020 ▸) in steels.

The grain grouping step is where a lot of methods deviate, with some codes reconstructing without grouping grains first, and others grouping prior to reconstruction. For example, the triplet method (Krishna *et al.*, 2010 ▸) uses the groupoid method (Cayron, 2006 ▸; Cayron *et al.*, 2006 ▸) to identify triplets of grains with a low tolerance angle. These ‘nucleus’ triplet grains are then grown by adding neighbouring grains that meet a tolerance condition, with this nucleation and growth cycle repeated until no more nuclei can be found. This is a non-iterative and quick method, but it may not group all of the grains if they do not meet the tolerance condition to be grouped together due to there being insufficient ‘nucleus’ triplets in the data set. Alternatively, there is the cluster method (Hadke *et al.*, 2016 ▸), which identifies clusters of the child phase that are highly likely to have originated from the same parent grain, then grows these clusters and back-transforms them to the parent phase using the summation of minimum misorientation angle method (Tari *et al.*, 2013 ▸). This is a computationally expensive iterative method with good accuracy.

Another potential issue is the artificial reunification of grains, particularly in cases where the grains are grouped prior to transformation. This can be addressed in some methods by adjusting tolerance values or coarsening parameters [*e.g.* for the Markov clustering (MCL) algorithm (Gomes & Kestens, 2015 ▸; Nyyssönen *et al.*, 2018 ▸)]. Similarly, uncertain grains (either at the grouping stage or to determine the parent orientation) are often an issue, either due to island grains within larger grains, or just small groups of child grains. These are dealt with in a number of ways, from not being reconstructed (Cayron *et al.*, 2006 ▸; Cayron, 2007 ▸), to additional subroutines in the code (*e.g.* Germain *et al.*, 2012 ▸, 2019 ▸) or back-filling from neighbouring grains (Krishna *et al.*, 2010 ▸).

For large data sets, there is a tension between accuracy and computational time, so some methods employ statistical analysis to support parent grain reconstruction. These include work by Glavicic *et al.* (2003*a* ▸,*b* ▸) which uses Monte Carlo analysis and two misorientation minimization steps and has the advantage of requiring no *a priori* knowledge of the parent grain boundary locations or variant selection. Another example is work by Gomes & Kestens (2015 ▸), Nyyssönen *et al.* (2016 ▸, 2018 ▸) and Nyyssönen (2020 ▸), which employs the MCL algorithm to create a network of discrete clusters of similar grains. This method is computationally efficient, has excellent noise tolerance and has only one coarsening parameter. Some groups have also worked to combine several of the above methods; for example, the work of Bernier *et al.* (2014 ▸), which brings together OR refinement (Nyyssönen *et al.*, 2016 ▸; Miyamoto *et al.*, 2009 ▸; Humbert *et al.*, 2011 ▸), local pixel-by-pixel analysis (Miyamoto *et al.*, 2009 ▸, 2010 ▸), and nucleus identification and spreading (Germain *et al.*, 2012 ▸).

In this paper, we first describe an algorithm based upon the parent austenite grain reconstruction code by Nyyssönen *et al.* (2016 ▸) using MCL, which we have adapted for two-phase body-centred cubic (b.c.c.)/hexagonal close-packed (h.c.p.) materials. In brief, this algorithm reconstructs the microstructures by identifying grain boundary types, then creating a discrete network of clusters of similar grains using a Markov chain cluster analysis of the initial microstructure. The BOR is then used with these clusters to reconstruct the parent grain microstructure. We demonstrate the reconstruction code using a simulated data set. Next, we apply the method to an experimentally obtained data set from Zircaloy-4 which has been quenched from the parent β phase. In this data set, we explore the relationship between parent β grain size and shared crystallographic features with regards to the phase transformations and mechanical properties.

## 2. Symmetry and crystallography of the BOR

The OR for the b.c.c./h.c.p. phase transformation in zirconium alloys [as reported by Burgers (1934 ▸)] is

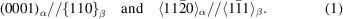




### 2.1. b.c.c. to h.c.p.

**Figure 1:**
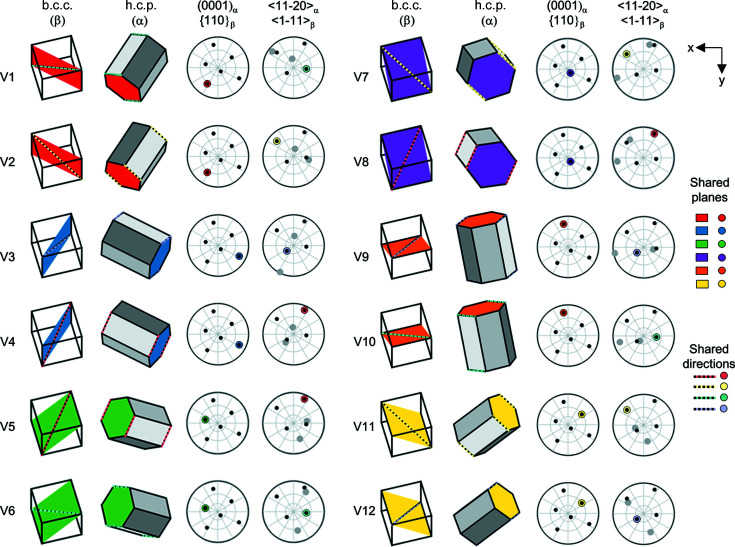
Twelve unique α variants (V1–12) for a fixed β orientation of (0, 30, 15) showing the corresponding planes and directions between the b.c.c. and h.c.p. orientations via prisms and pole figures. For the prisms, shared planes between variants are indicated by the colour of the plane, and shared directions between variants are indicated by the coloured and dashed directions. For the pole figures, the shared plane or direction is shown by the coloured dot, the other h.c.p. points by the larger grey dots and the b.c.c. orientation by the smaller black dots.

For any initial β grain, the transformed α orientation can be one of 12 unique variants, as described by the BOR, and this is shown in Fig. 1 ▸.

In Fig. 1 ▸, the β phase (b.c.c.) orientation is kept constant, with each of the six 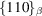 planes highlighted in turn, in a different colour. Each of these 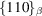 planes contains two different 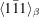 directions, *e.g.* for 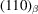 , the two directions are 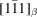 and 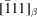 . For each cube shown, one of the two 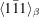 directions contained within has been highlighted with a dashed pastel coloured/black line. The corresponding hexagonal prisms (α orientations – which do change) are shown in the columns adjacent to the cubes. In each case, the 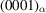 plane is highlighted with the same colour as the plane that it matches in the b.c.c. orientation and the 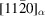 direction has the same pastel coloured/black dashed line as its corresponding 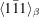 direction in the b.c.c. orientation. This information is shown on the pole figure plots for the b.c.c./h.c.p. planes and directions. In this case, the b.c.c. orientation (including symmetry) is shown with the small black dots, and the larger dots are the h.c.p. planes and directions. The overlapping, coloured point is the shared plane or direction and the colours correlate with the prisms. Note that all prisms and pole figures have the same axes.

As can be seen in Fig. 1 ▸, there are common shared planes and directions between the 12 unique α variants that can result from an initial parent grain orientation. There are four groups of variants that share the same shared direction, as shown by the colour of the directions in the figure, *e.g.* V1, V6 and V10 have a common shared direction. Similarly, there are six pairs of α variants with the same shared plane, *e.g.* V1 and V2.

### 2.2. h.c.p. to b.c.c.

**Figure 2:**
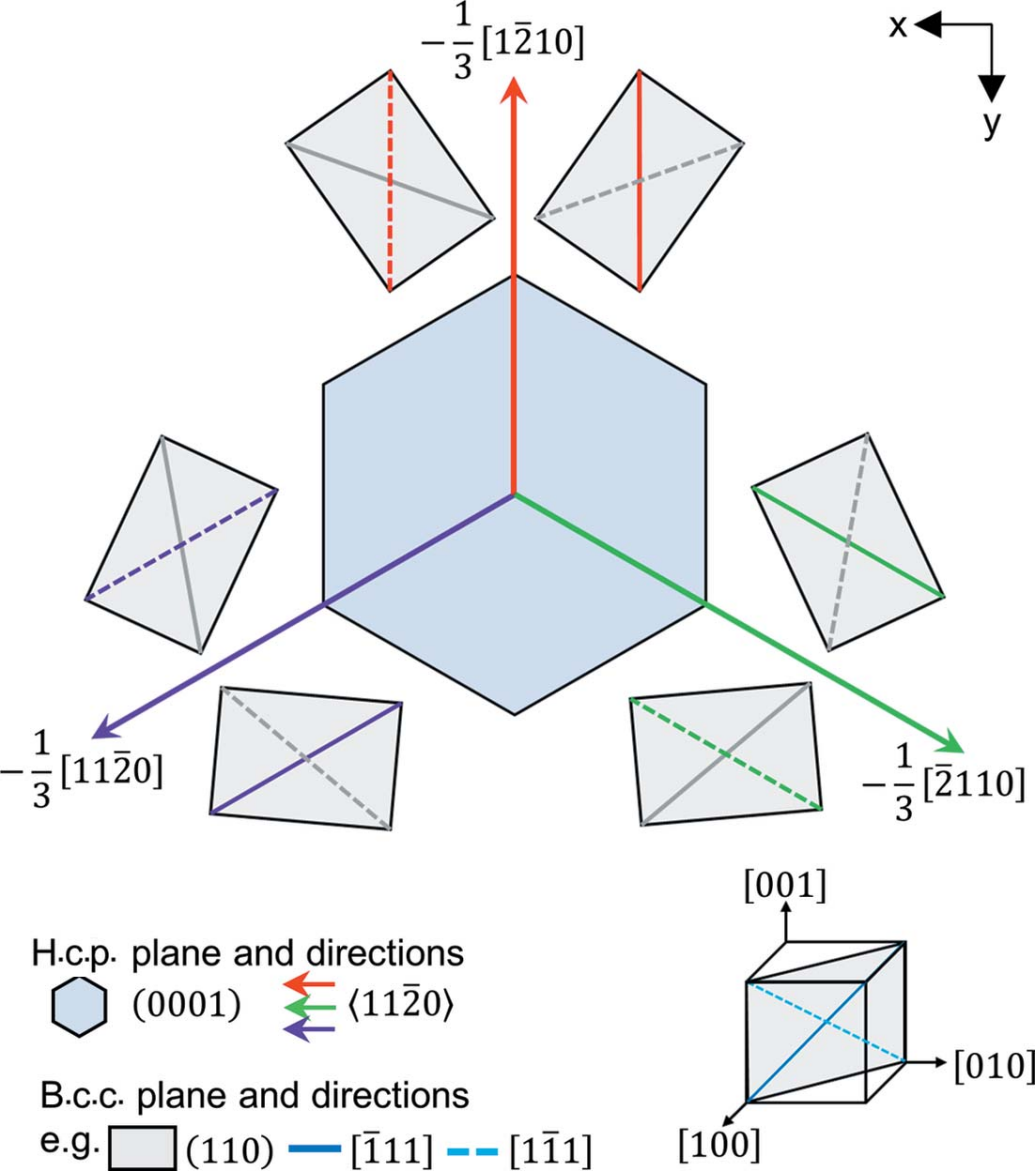
Six potential unique parent β orientations for a start α reference orientation. Parallel directions are indicated using the 〈*a*〉 direction colouring.

When the situation is reversed, any start h.c.p. orientation gives rise to six potential unique b.c.c. orientations, as illustrated in Fig. 2 ▸. The h.c.p. crystal is aligned with the 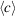 axis out of plane and one 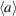 direction pointing upwards, as per our chosen convention of the reference crystal orientation for h.c.p. For each 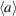 direction parallel with a 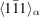 in the b.c.c. phase, there are two orientations of the b.c.c. crystal. This can be explained as follows: for each h.c.p./b.c.c. orientation alignment, such that we align 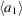 (which is parallel to 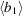 in the β phase) as a common 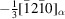 , then for each of the two related variants the 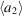 and 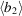 relationship (which is 10.52° misaligned) switches from 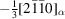 to 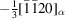 . Six variants in total are generated, as this is repeated three times for each of the three h.c.p. 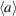 directions.

## 3. Reconstruction algorithm and microstructural post-processing

We have developed an *MTEX* (Bachmann *et al.*, 2010 ▸)-based code in MATLAB (see Section 11). This code can be broken down into two sections: initial reconstruction and post-processing.

### 3.1. Initial reconstruction

This section of the code is adapted from work by Nyyssönen *et al.* (2016 ▸, 2018 ▸) and Nyyssönen (2020 ▸). The existing algorithm is used to reconstruct the parent austenite microstructure from a child martensite microstructure found in steels. To adapt this code for the b.c.c. to h.c.p. (β→α) phase transformation in h.c.p. alloys, both the phases and OR have been updated (which includes consideration of the reference orientations of the α and β phases). Here we follow the conventions described by Britton *et al.* (2016 ▸) where the *b* axis of the reference h.c.p. unit cell is aligned along the *b* axis of the reference b.c.c. unit cell. We use the BOR described in equation (1).

As we have discussed, using this OR, there are 12 potential unique α variants for any start β orientation. In reverse, there are six potential unique β variants for any start α orientation (Karthikeyan *et al.*, 2006 ▸). This is important in the initial reconstruction and post-processing algorithms.

**Figure 3:**
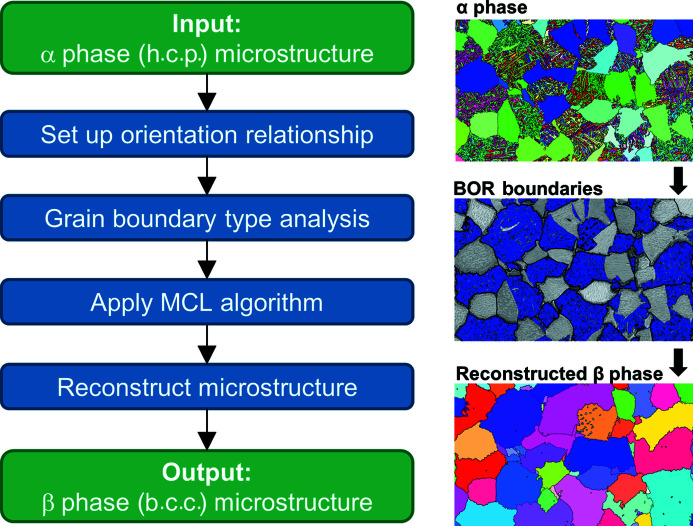
Initial reconstruction code overview – steps to go from the input α phase microstructure (h.c.p.) to the reconstructed β phase (b.c.c.) microstructure, with microstructure schematics.

The algorithm for the initial reconstruction section is shown schematically in Fig. 3 ▸ and contains four main steps: (i) setting up the OR; (ii) identifying grain boundary types; (iii) applying the MCL algorithm; (iv) reconstructing the parent β microstructure.

#### 3.1.1. Setting up the OR

Firstly, the OR is established for the β→α transformation. The transformation matrix ( 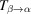 ) is set up using an initial pair of orientations [ 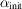 (0, 45, 24.7) and 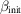 (0, 0, 0)] that meet the BOR. The BOR relationship links the b.c.c. to h.c.p. orientations, and describes how one of the 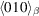 directions matches to the h.c.p. unit cell. Different EBSD indexing algorithms change these relationships. We follow Britton *et al.* (2016 ▸) with 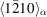 // **Y** and 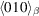 // **Y** for the reference orientations (note that Britton *et al.* use a convention where 〈*b*〉 is parallel to the *Y* axis, and in the h.c.p. unit cell 〈*b*〉 is one of the 〈*a*〉 directions):

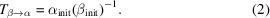

This is then expanded to include symmetry:

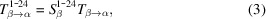

where 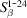 are the 24 symmetry matrices for the b.c.c. phase. This allows the potential α variants ( 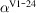 ) to be calculated for any start β orientation (β) using

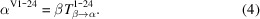

Note that, due to symmetry, only the first 12 are unique, the second 12 are repeats.

#### 3.1.2. Identifying grain boundary types

The OR is then used to identify parent grain boundaries on the α grain map. A set of α variants is calculated and the misorientations between the variants found. Due to the symmetry, the list of misorientations can be minimized to a short list of angle/axis pairs (Gey & Humbert, 2003 ▸; Karthikeyan *et al.*, 2006 ▸) as detailed in Table 1 ▸.

It is these angle/axis pairs that are used to evaluate whether each grain boundary segment was a parent grain boundary or not. In the experiment, the misorientation across an α–α boundary may not be exact, due both to measurement uncertainty and rotation away from transformation strain. A threshold of 4° is set as the default. Boundaries which are not described by the special relationships in Table 1 ▸ (within the selected threshold) are classified as non-BOR, *i.e.* typically a parent grain boundary.

#### 3.1.3. Applying the MCL algorithm

At this point, the MCL algorithm (van Dongen, 2000 ▸) is applied to identify discrete clusters of closely related α grains that will be used for the reconstruction step. The aim of using a clustering algorithm is to quickly identify the child grains that originated from the same parent grain and speed up the reconstruction process. In brief, the MCL algorithm enables groups of related BOR α grains to be clustered together for parent β orientation determination. Similar approaches exist (Hadke *et al.*, 2016 ▸; Germain *et al.*, 2012 ▸), but often require further steps to incorporate grains not included in the initial analysis (due to threshold conditions).

The algorithm creates a network based upon the closeness of grain boundaries to the potential grain boundaries. This network is then put through a series of expansion and inflation steps until a discrete network of clusters is established. For more discussion of the MCL algorithm, see Nyyssönen *et al.* (2018 ▸). An advantage of this algorithm is the single factor for adjusting it – the ‘inflation power’. In practice, this term can change the number of parent β grains that are found. A smaller inflation power decreases the number of discrete clusters identified, *i.e.* the user is advised to explore variation of the inflation power and the output reconstruction (which is assisted by the post-processor, described later).

#### 3.1.4. Reconstructing the parent β microstructure

Once clusters of α grains are joined together to describe one parent β grain, it is valuable to determine the parent β grain orientations. The reconstruction step combines (i) and (iii), with each discrete cluster of α grains treated individually. For each α grain within the cluster, the potential β grain orientations are calculated (using 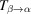 ). When this is completed for every α grain within the cluster, a density function is used to find the most intense orientation – this is allocated as the β orientation for that cluster. Once this is completed for all discrete clusters, an EBSD map for the parent β phase can be plotted. Our code then processes the grains for the β phase ready for the post-processing section.

### 3.2. Post-processing

Once the parent β grain boundaries are determined, it is possible to evaluate (i) the uniqueness of the determined β orientation; (ii) the shared 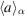 directions within the parent β grain; and (iii) the shared 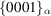 planes within the parent β grain.

The additional information generated in this section of the code can then be used to look at the influence of grain boundary types and variant selection.

#### 3.2.1. Certainty of the β orientation and the associated α variants

The α variants are calculated on a grain-by-grain (β) basis – this allows for comparison of α variants within each β grain, but not between (β) grains. The process used to analyse the α variants present begins by setting the β grain orientation to the fundamental zone ( 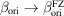 ) and then using this to calculate the potential α variants ( 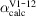 ):

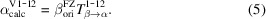

The minimum misorientations between each of the calculated α variants and the parent β orientation ( 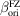 ) are then calculated and stored as the ‘ideal’ misorientations:

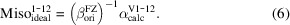

Next, the experimental β→α misorientations ( 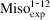 ) are calculated for each α grain present ( 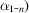 within the parent β grain ( 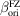 ):

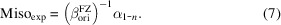

Finally, each experimental misorientation is compared with the list of ideal misorientations – the number of the closest match is taken to be the α variant ID for that grain.

#### 3.2.2. β certainty

The number of potential β orientation options (β certainty) for each parent grain identified depends on the number and type (whether there are shared planes or directions) of unique α variants present. For example, if there is only one α grain, it is not possible to determine which of the six potential β orientation options is the parent β orientation.

**Figure 4:**
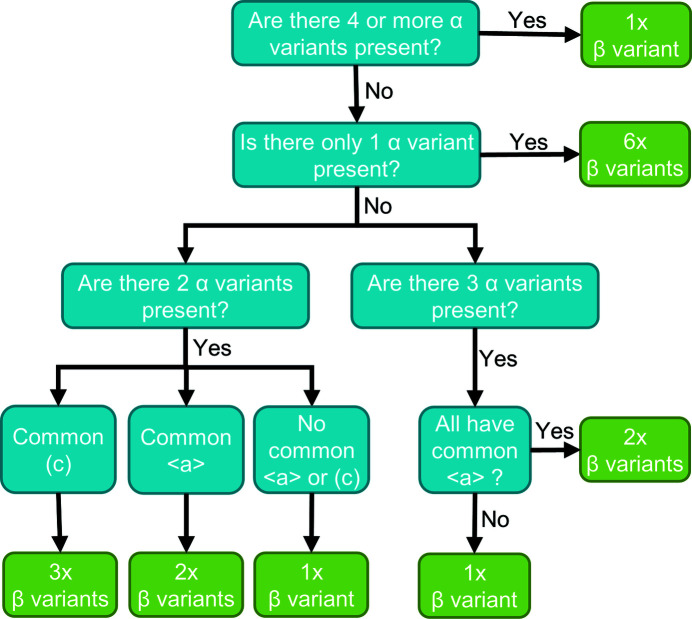
Decision flowchart for the number of potential unique parent β grain orientations, depending on the number and type of unique α variants present.

To fully determine the parent β orientation, the number of unique α variants required depends on the type of variants present [shared basal plane, which we call (*c*) in this paper; or shared 〈*a*〉 direction; or neither]. In the case where there is a shared plane, three of the β options will be the same, whilst for a shared direction, two options will be the same between variants. The minimum number of unique α variants required for a unique β parent is two and the maximum is four in the case where three of the variants have a common 〈*a*〉 direction. The algorithm used for determining the number of β options is described in Fig. 4 ▸.

#### 3.2.3. β orientation options

As part of the step to calculate the β certainty, all potential parent β orientation options (β options) are stored, as calculated from the associated α grains within the parent β grain. For uniquely determined β grains, this is a single orientation, but where there is uncertainty different orientations can be represented. Additionally, the number of potential β orientations can be reported.

#### 3.2.4. Analysis of the structural units within the α phase: shared planes and directions

With the α variants identified for each parent β grain, variants with shared planes or directions can be identified. Within the set of 12 unique α variants, there are a number of combinations that share a common plane or direction, as illustrated in Fig. 1 ▸ for a set of α variants calculated from a start β orientation of (0, 30, 15). This enables the α grain map to be plotted coloured by variant number (coded with respect to each parent β grain), as well as the shared 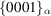 planes, or shared 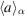 directions within each parent β grain. Analysis of the relationship between the α phase domains within the parent β grain is similar to the analysis of parent austenite grain block and packet analysis, as explored by Morito *et al.* (2003 ▸). Note that the variant numbering for α domains may be different within two adjacent parent β grains.

## 4. Simulated data set

**Figure 5:**
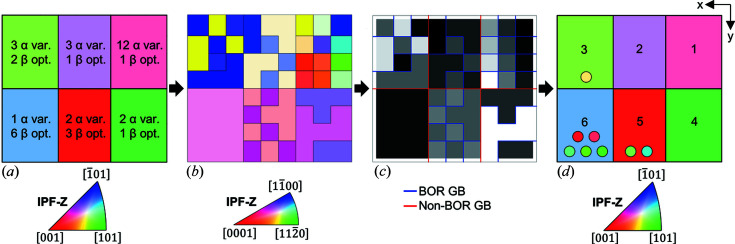
Simulated data set in (*a*) β phase (IPF-Z) and (*b*) α phase (IPF-Z). (*c*) Grain boundaries identified by type on a greyscale variant number background: red = parent grain boundary, blue = α/α grain boundary. (*d*) The reconstructed parent grain microstructure (IPF-Z) – where there are multiple β grain orientation options, the alternative orientations are shown in the circles overlaid on the grains. The grain numbers on (*d*) correspond to those reported in Table 2 ▸.

To validate the algorithm, a simulation was performed. A data set consisting of six β grains was created with a ‘checkerboard’ morphology to test whether the algorithm would determine the exact parent β microstructure and whether the uncertainty was correctly evaluated. This data set is shown in Fig. 5 ▸.

From this β grain structure, an α microstructure was then created by assigning an α variant number to each of the 12 sub-grains contained within each parent grain.

To test varying the number of unique α variants present and the effect of shared planes or directions, the assigned α variant numbers were carefully selected for the simulations for each parent grain, as detailed in Table 2 ▸. The potential α variants for each parent grain orientation were then calculated and the appropriate orientation assigned to each α sub-grain. The resultant α microstructure was used as the input for the algorithm.

The simulated data set and the corresponding reconstructed parent β microstructure are shown in Fig. 5 ▸ (and are provided as supporting data). As can be seen in Fig. 5 ▸(*c*), the parent grain boundaries are correctly identified (red boundaries), as are all of the α–α boundaries within each grain (blue boundaries). The reconstructed parent grain microstructure is correct for each of the ‘known’ (one potential β orientation) parent grains. However, as expected, there is uncertainty for the remaining grains. The chosen parent grain options can be selected to match the original parent β orientations, but the output may not select this (as there is a random selection of the represented β grain from the uncertain candidate orientations). For each uncertain grain, the alternative β orientation options are presented as the circles within each grain in Fig. 5 ▸(*d*).

## 5. Experimental materials and method

For the experimental example, a Zircaloy-4 sample with a grain size of ∼11 µm was used [from the same batch of materials as used by Tong & Britton (2017 ▸)]. The sample was encapsulated in a quartz tube, backfilled with Ar, heated to >1000°C (*i.e.* super-transus) for 10 min and then water quenched. The sample was then ground using 800–2000 grit SiC papers and polished for 4.5 h with OP-S. Finally, the sample was broad ion beam polished using a Gatan PECSII machine for 15 min at 8 keV, 8° tilt, 1 r min^−1^, and with dual modulation.

EBSD data were captured using a Quanta FEG 650 scanning electron microscope equipped with a Bruker eFlashHD2. Data were processed using Bruker *ESPRIT* v2.2 software and exported to .h5 prior to analysis in MATLAB (R2018b) using *MTEX* version 5.4.0. EBSD patterns were captured using a 20 kV beam with a working distance of 15 mm and 70° sample tilt. The patterns were captured at a resolution of 320 × 240 pixels using a 34 ms exposure time and 0.14 µm step size.

## 6. Results

**Figure 6:**
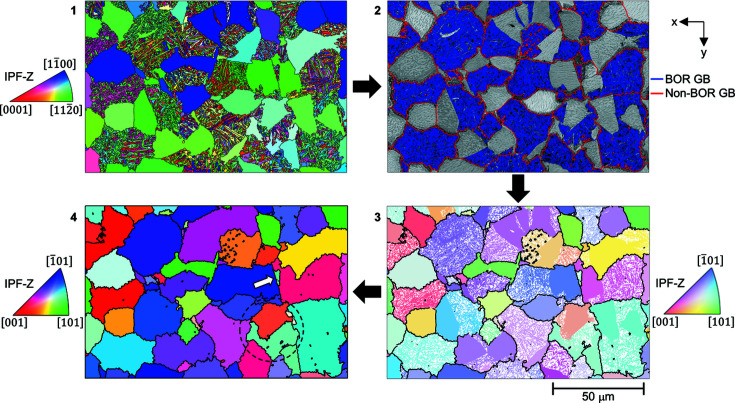
Reconstruction of an EBSD map from a β quenched Zircaloy-4 sample showing (1) initial α microstructure (IPF-Z); (2) pattern quality map with overlaid grain boundaries, identified by type: red = parent grain boundaries; blue = α–α (or BOR) grain boundaries. (3) Initial reconstruction output – β phase (IPF-Z); (4) reprocessed parent β grains (IPF-Z) with an arrow showing the location of the grain selected for further analysis. The grain pair within the dashed circle is explored in Appendix *A* [App appa].

The reconstruction of the experimental data is shown in Fig. 6 ▸, which includes the α orientations, the grain boundary map with labelling of the BOR boundaries and associated parent β microstructures. For the parent β microstructures, the initial reconstruction output [Fig. 6 ▸(3)] is based on the α grain groupings identified using the MCL algorithm – the grain boundaries present are the non-BOR boundaries identified at step (ii). The reprocessed map [Fig. 6 ▸(4)] takes the orientations from the initial reconstruction map and processes the grains.

This microstructure has (large) globular α domains, which have an approximate diameter of 20 µm. The origins of these globular domains are unclear. The microstructure we observe here is similar to many Ti-6 Al-4 V microstructures (Warwick *et al.*, 2013 ▸), where the globular α grains are thought to originate due to recrystallization of α laths in the α+β phase field. In our experiment, this globular α nucleation and growth could occur as the sample is taken from the furnace and dropped into the quenching fluid. While the precise mechanism of this process is beyond the scope of the present work, we note that often these globular grains have an orientation that is related to the neighbouring α lath region.

Between these globular regions, there is a fine-scale secondary α lath microstructure with multiple different α variants. The size and shape of these regions containing secondary α regions are of similar size to the globular α, but the α laths tend to have a width of ∼1 µm. Analysis of the grain boundary map shows that the globular α and variants of α within the transformed secondary α domains are often from the same parent β orientation, and this is shown within the reconstructed parent β orientation maps in Fig. 6 ▸.

An artefact was revealed in this reconstruction, as highlighted by the dashed circle in Fig. 6 ▸ stage (4). In the reconstruction shown, this is labelled as two parent β grains. However, in this reconstruction, the parent β grain boundary has a shared 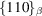 and this explains why adjustment of the inflation power and cut-off tolerance angle can result in this region being reconstructed as a single (larger) parent β grain. For more information on this analysis, see Appendix *A* [App appa].

**Figure 7:**
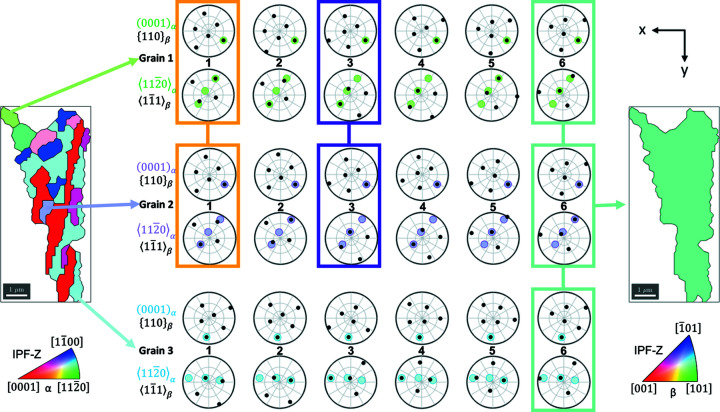
Example showing how the β orientation certainty is affected by the number of unique α variants present. There are three potential β variants (shown by the coloured and linked boxes) with just α grains 1 and 2, whereas with all three grains this reduces to one orientation (β variant 6 for all grains). α grain orientations are shown by the coloured dots in the pole figures, whilst the six potential β orientations for each of these are shown by the black dots in the pole figures.

To explore the certainty of the parent β reconstruction, one region was extracted from the map in Fig. 6 ▸(4) and the ORs are plotted in Fig. 7 ▸. In this example, the α phase structure includes eight variants of α phase, and so the parent β orientation is overdetermined. However, we can analyse the stereographic relationships between the α variants and parent β orientation to show which combinations of α variants are required to determine a unique parent β orientation.

Three variants are highlighted, with the stereographic projections of the final (*i.e.* correct) β and α phase crystal planes and directions which are involved in the BOR. The combination of grain 1 (light green) and grain 2 (lilac) results in three potential parent β crystal orientations, as they have a shared basal plane. This ambiguity is immediately resolved when a third variant is identified from the same parent β grain if this (new) variant does not share the same basal plane.

Examination of the other five α orientations (not shown in the figure) confirms that the β orientation determined is consistent with all the child α variants found in this region.

## 7. Post-processing

The parent β reconstruction algorithm enables us to explore the relationship between the α grains within the complex microstructures. As we have demonstrated that the parent β microstructure can be determined using this algorithm, we can use it to explore the relationship between microstructural units within the α phase map.

In terms of mechanistic understanding, it is useful to report on four relationships:

(i) The number of potential parent β orientations, *i.e.* the uniqueness of the parent β reconstruction.

(ii) A labelled map of each α variant within each β grain.

(iii) Sharing of the basal plane within each parent β grain.

(iv) Sharing of the 〈*a*〉 direction within each parent β grain.

**Figure 8:**
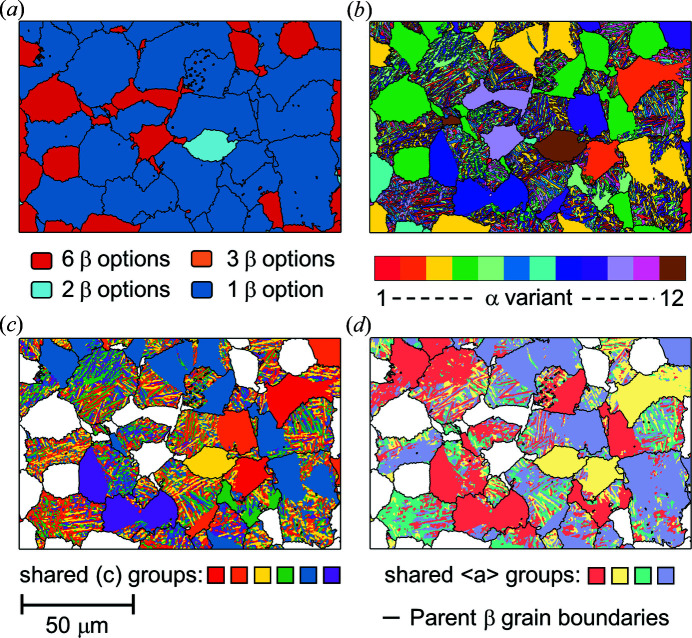
Post-processing outputs for β quenched Zircaloy-4 samples. (*a*) β orientation certainty map showing the number of potential β variants for each parent β grain. (*b*) α variant map – all 12 variants shown. (*c*) α variant map coloured by α variants with shared (*c*) planes. (*d*) α variant map coloured by α variants with shared 〈*a*〉 directions. Note: for (*b*)–(*d*) comparisons can only be made directly within the grains as the maps are calculated on a grain-by-grain basis.

For example, four maps for these relationships (using the experimental data set) are shown in Fig. 8 ▸. These maps are related to each other, and can be insightful depending on the microstructural analyses required.

Uncertainty of the parent β orientation is dependent on the number of α variants generated from each parent β, combined with whether these variants share 〈*a*〉 directions and/or basal planes. This is shown pictorially in Fig. 7 ▸. This ambiguity was also explored within the simulated data set shown in Fig. 5 ▸. The uncertain cases, and the reason why they are uncertain (*e.g.* due to shared basal planes or shared 〈*a*〉 directions) can be inferred from Fig. 8 ▸, such as for the pale-blue region [in Fig. 8 ▸(*a*)] where there are two β options. In this case, there are two α variants present (the second grouped variant is a smaller grain not visible on the figure) which share a common 〈*a*〉, hence the ambiguity.

The map of variants shown in Fig. 8 ▸(*b*) can be useful to evaluate the size and shape distribution of variants within a parent β grain. For example, where the large globular α and lath α reconstruct to the same parent grain, the globular α variant type and the fine-scaled transformed α can be compared (different or similar variants). The colour coding as presented plots the variants with respect to each parent β grain structure, as the relationship between α variant numbering from one parent β grain to the next cannot be read.

The map of shared basal planes is likely to be useful when considering properties that will extend across microstructural regions, such as any property that is transversely isotropic in the zirconium system (*e.g.* elastic stiffness). This map could also be useful to help navigate arguments in the literature on failure under fatigue loading and the formation of facets (Uta *et al.*, 2009 ▸). Again, similar to the variant analysis, this map must be interpreted with respect to the parent β grain structure, as a similarly coloured shared basal plane in this map across two β grains is unlikely to have a common plane normal.

Finally, the map of shared 〈*a*〉 directions echoes the idea of the shared basal plane map, but now is true for the 〈*a*〉 directions. This map could be important when considering the propagation of slip through the microstructure and the relative transparency of different α/α grain boundaries. Furthermore, if there is a transformation strain associated with the β→α OR, potentially analysis of this map could assist in understanding transformation strain heterogeneity and variant selection (Shi *et al.*, 2016 ▸).

## 8. Comparison with *MTEX*

In the process of developing our algorithm, a reconstruction algorithm was developed by the creators of *MTEX*.

We followed their tutorial and performed a reconstruction using *MTEX 5.7.0*. The following recipe was used:

Calculate (α) grains (4° threshold used for direct comparison), remove quadruple points and remove small grains (less than five points).

Compute parent orientations from triple junctions (and probabilities).

Use a voting system (threshold 0.7) to determine which grains are converted to parent grains.

Grow parent grains at grain boundaries.

Merge parent grains.

Merge inclusions.

**Figure 9:**
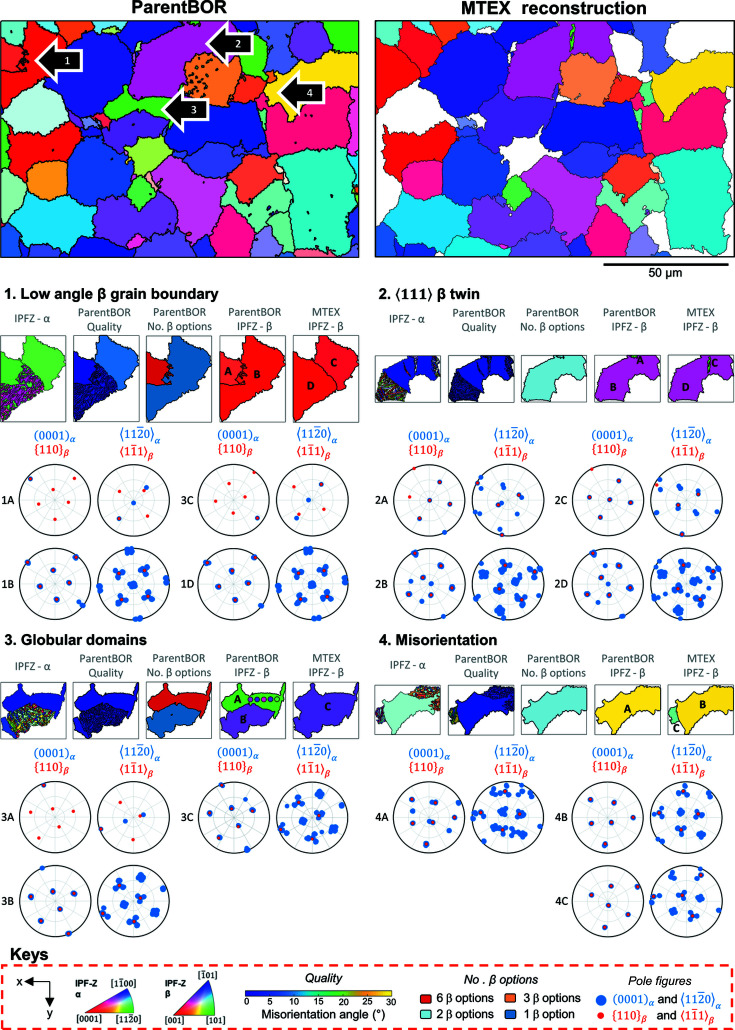
Comparison of *ParentBOR* reconstruction and *MTEX 5.7.0* reconstruction.

A comparison of the reconstruction between the *ParentBOR *code and the *MTEX* reconstruction is shown in Fig. 9 ▸. Of note, the *MTEX* reconstruction algorithm does not include any of the post-processing analysis that we have described for the *ParentBOR* code. In practice, this could be included in the *MTEX* algorithm but it would need some subtle (but important) changes with respect to how the variants are numbered and how the results are described within the *MATLAB* variable arrays.

The *MTEX* reconstruction does not reconstruct regions where there is more than one parent β grain orientation, and these are shown as white regions in Fig. 9 ▸. In addition to these (apparently unsolved) regions, there are four other types of difference between the two reconstructions.

### 8.1. Type 1 – low-angle β grain boundary

The *MTEX* reconstruction and the *ParentBOR* reconstruction differ in how they build up the parent grains. In the *MTEX* reconstruction, variant orientations are considered with respect to triple junctions. In contrast, in the *ParentBOR* reconstruction domains are reconstructed using the MCL algorithm. In region 1, the large ‘red’ grain (*i.e.* near [001] out of plane) is broken down with two different sub-grain boundaries. In practice, these regions are closely aligned, and they could be post-processed to be joined together within the same large region of parent β structure.

### 8.2. Type 2 – 〈111〉 β twin

There is a small region within the pink grain which has a different orientation (in the parent reconstruction) to the neighbourhood. Analysis of the β pole figures reveals that this region is a 〈111〉-type twin in the parent β orientation. The OR between these two regions in the β phase, and the associated inheritance of this OR through the α domain, results in a slight confusion in the size and extent of the parent β grain size (and the very small grain within this parent β domain).

### 8.3. Type 3 – uncertainty of globular domains

The MCL clustering regime tends to grow domains outwards from clusters of similar orientation. Spatial regions which include a single variant can cause issues when the reconstruction is performed. Here we see that there is a region (A in Fig. 9 ▸) which does not have a certain parent β orientation, but the candidate orientations of this region include both the green (the ascribed colour) and the purple orientation (closely aligned to region B, and similar in orientation to the *MTEX* domain, labelled C). This highlights that the *ParentBOR* code could be extended further to post-process the parent β map, for regions that are less certain, to consider whether they belong to nearby regions.

### 8.4. Type 4 – misorientation

For this region, we observe that the *ParentBOR* reconstruction shows one parent β grain and the *MTEX* reconstruction reveals two separate parent β domains. Analysis of the pole figures shows that region A (the *ParentBOR* grain) has the same β orientation as region B (the large area from the *MTEX* reconstruction). In region A, however, there is an area which has a high angular deviation for the left-hand shoulder (which *MTEX* describes as a different β grain). The poor reconstruction of this area is shown as a high value (*i.e.* bad) in the quality map. It is likely that the MCL algorithm clusters these two regions together despite there being a small misorientation between the α variants (and associated small angular deviation for the β grains).

## 9. Discussion

We present an evolved form of parent β grain analysis, together with some new post-processing capabilities.

The MCL algorithm to group the α grains prior to reconstruction provides a good balance of speed and accuracy, whilst the single coarsening parameter (inflation power) provides the opportunity to easily adjust the number of discrete clusters identified. This has benefits and disadvantages, as there is the potential to add parent grain orientations if too many clusters are identified. The inflation variable can average small sub-groups of grains within each parent, and this risks adding ambiguity to the analysis. This is also implemented within *MTEX*, and released as open source, which may promote wider use and further developments of the method.

In the reconstruction, we have chosen to use all α grains present for our code, but this leads to uncertain parent grain orientations in cases where there are insufficient variants present. The post-processor for the approach will subsequently label these regions and provide suggestions for the alternative β orientations which may be selected, as per users’ needs.

The simulated data set (with code available as supporting information) allows us to validate the reconstruction and test the post-processing, and it can be adapted to different sized data sets. In development of our approach, the simulation has been useful to explore specific scenarios, *e.g.* where there is uncertainty as to why a parent β grain reconstructs as two grains or an α grain flips between two parent β grains with changing inflation power. Application of the code to experimental data revealed further nuances, such as the ORs within the parent β structure that can cause confusion within the reconstruction.

We have also evaluated the *ParentBOR* code against recently available code within *MTEX* (see Section 8). While there are (understandable) differences between these reconstructions, it is also useful that we now have two analysis methods that are constructed within the same overall framework. This enables us to understand how reasonable (or unreasonable) the reconstruction is with regards to specific algorithm-induced artefacts. The relevance of these artefacts will depend on how the parent β grain structure is interrogated. This comparison highlights that the *ParentBOR* code reconstructs a significant fraction of the map similarly to the *MTEX* implementation, and that there are specific regions which are reasonably reconstructed in *ParentBOR* and vice versa. This implies that a hybrid approach could be implemented, such as the recently described methods of Huang *et al.* (2020 ▸) which include OR refinement, orientation coalescence and regional voting.

Recent advances using pattern matching by Lenthe *et al.* (2020 ▸) of overlapped BOR-related patterns offer the chance to index finer-scaled microstructures, where there may be two or more overlapping patterns within the interaction volume. Fortunately, our microstructure mapped sufficiently well (>83% success) and there is no retained β in this material.

From a materials engineering perspective, we expect that the post-processing analysis is likely to prove useful for understanding variant selection during thermomechanical processing of alloys with a b.c.c.-to-h.c.p. allotropic phase transformation, such as titanium, zirconium and hafnium. For the future, it may also be useful to explore the relative effectiveness of different grain boundaries for specific materials properties, such as the effective slip length during deformation.

## 10. Conclusion

In this work, we have (i) introduced a h.c.p.→b.c.c. parent phase reconstruction method and demonstrated this on simulated and experimental zirconium material data; (ii) created a post-processor to reveal the uniqueness of the parent β grain orientations and shared crystallographic relationships within the parent β grains.

We found that the MCL method can be adapted to different ORs successfully, and the addition of a post-processing step (specific to the materials system) can be useful to understand associated ambiguity of the parent microstructure. We hope that the post-processing tools, which have been designed to explore shared basal planes and shared 〈*a*〉 directions, will be useful in understanding materials performance issues in engineering applications.

## 11. Data availability

To further work in this field, we also provide our algorithms and data. The *ParentBOR* analysis code is available via https://github.com/ExpMicroMech/ParentBOR and the example data can be found via Zenodo (https://doi.org/10.5281/zenodo.4632039).

## Supplementary Material

Example data set: https://doi.org/10.5281/zenodo.4632039

## Figures and Tables

**Figure 10:**
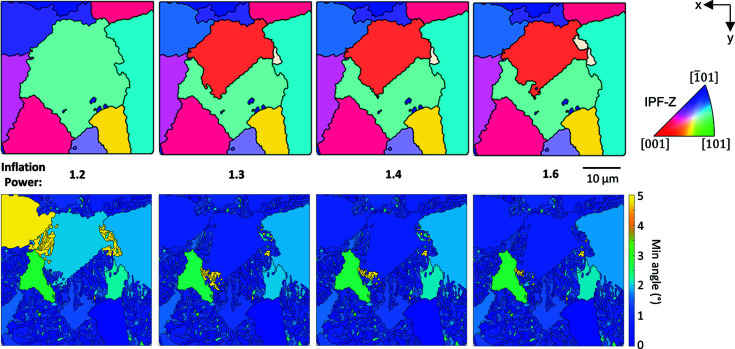
Reconstruction of a smaller region into one or two (main) parent β grains, showing the effect of changing inflation power from 1.2 to 1.6. In this figure, the upper row shows the reconstructions as coloured using the IPF-Z colour map, and the bottom row shows the calculated misorientation of the (as-measured) α grains from the ideal α orientations determined from the parent β grain orientation.

**Figure 11:**
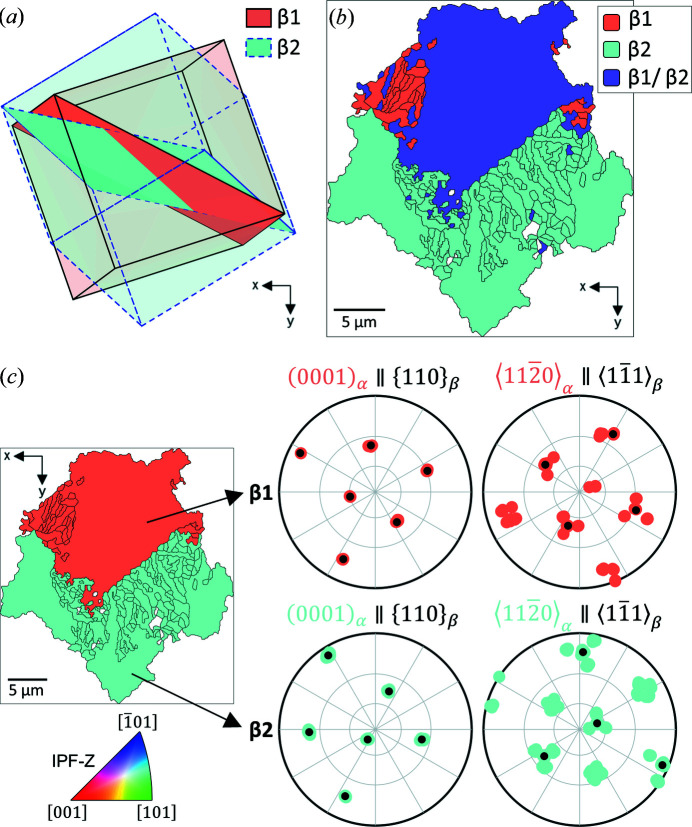
(*a*) Comparison of the two β grain orientations, showing the shared planes for each using solid colour (corresponding to the IPF-Z map colours for each grain). (*b*) α grains for this region coloured by parent grain orientation β1, β2 or uncertain (β1/β2). (*c*) Manually sorted α grains, coloured by parent β grain assigned (IPF-Z colouring) – along with the pole figures for each parent β grain region, with the α orientations shown by the coloured dots and the β orientations by the black dots in each case.

**Table 1:** Minimized list of angle/axis pairs for α-zirconium that can exist between α variants from the same parent β grain As determined from our code, but similar to work reported by Gey & Humbert (2003 ▸) and Karthikeyan *et al.* (2006 ▸).

		Axis			
ID	Angle (°)	*h*	*k*	*i*	*l*
1	10.53	0	0	0	1
2	60	−1	2	−1	0
3	60.83	−12	7	5	3
4	63.26	−2	1	1	1
5	90.00	−7	12	−5	0

**Table 2:** Simulated data set α variant population choices and the number of potential parent β orientations that can be found based on these

Grain No.	No. of unique α variants	Details	No. of potential parent β orientations
1	12	All 12 variants present	1
2	3	Two common 〈*a*〉 + one random	1
3	3	All common 〈*a*〉	2
4	2	No commonality	1
5	2	Both common (*c*)	3
6	1	Only one variant	6

## Appendix A

The parent β grain identified by the dashed circle in Fig. 6 ▸(4) can be reconstructed as a single parent β grain, or two (main) parent β grains. This uncertainty is caused by a closely aligned  plane between the two parent β grains as shown in this case. Whether it reconstructs as one or two grains depends on the number of discrete clusters formed prior to reconstruction – in other words, the inflation power. This is shown in Fig. 10 ▸ where a smaller region from the larger map has been selected and reconstructed using a range of inflation powers.

Looking at the two β grain options, the two orientations have a shared  plane, with a 1.34° angle between the two  planes and a total misorientation of 47° between the two crystals (*i.e.* they are rotated about the shared  ). This is shown visually in Fig. 11 ▸(*a*) where the two parent grain orientations have been plotted as overlapping cubes, with the common planes indicated by the solid colours. The consequence of this shared plane is that there are α variants which would match either parent grain orientation – this is where the ambiguity in the reconstruction occurs.

A manual analysis of the α grains contained within this region, in Fig. 11 ▸(*b*), shows the issue more clearly, with uncertain grains shown in purple. The region could plausibly be reconstructed as a single parent grain, although there would be regions [orange grains in Fig. 11 ▸(*b*)] that do not match this parent grain well – these could potentially be other parent β grains. However, a better reconstruction (comparing the α and β orientations on pole figures) can be achieved using the split grain option, as demonstrated by Fig. 11 ▸(*c*). It is therefore likely that the two parent β grains, with a shared  plane, is a valid reconstruction.
